# Deep-UV to Mid-IR Supercontinuum Generation driven by Mid-IR Ultrashort Pulses in a Gas-filled Hollow-core Fiber

**DOI:** 10.1038/s41598-019-39302-2

**Published:** 2019-03-14

**Authors:** Abubakar I. Adamu, Md. Selim Habib, Christian R. Petersen, J. Enrique Antonio Lopez, Binbin Zhou, Axel Schülzgen, Morten Bache, Rodrigo Amezcua-Correa, Ole Bang, Christos Markos

**Affiliations:** 1 DTU Fotonik, Technical University of Denmark, Kgs., DK 2800 Lyngby, Denmark; 2 CREOL, The College of Optics and Photonics, University of Central Florida, FL 32816 Orlando, USA

## Abstract

Supercontinuum (SC) generation based on ultrashort pulse compression constitutes one of the most promising technologies towards ultra-wide bandwidth, high-brightness, and spatially coherent light sources for applications such as spectroscopy and microscopy. Here, multi-octave SC generation in a gas-filled hollow-core antiresonant fiber (HC-ARF) is reported spanning from 200 nm in the deep ultraviolet (DUV) to 4000 nm in the mid-infrared (mid-IR) having an output energy of 5 μJ. This was obtained by pumping at the center wavelength of the first anti-resonant transmission window (2460 nm) with ~100 fs pulses and an injected pulse energy of ~8 μJ. The mechanism behind the extreme spectral broadening relies upon intense soliton-plasma nonlinear dynamics which leads to efficient soliton self-compression and phase-matched dispersive wave (DW) emission in the DUV region. The strongest DW is observed at 275 nm which corresponds to the calculated phase-matching wavelength of the pump. Furthermore, the effect of changing the pump pulse energy and gas pressure on the nonlinear dynamics and their direct impact on SC generation was investigated. This work represents another step towards gas-filled fiber-based coherent sources, which is set to have a major impact on applications spanning from DUV to mid-IR.

## Introduction

The ultraviolet (UV) spectral range is of great scientific and technical interest, mainly because a majority of molecules have strong electronic bandgap absorption bands in this region^1^. The mid-IR on the other hand is directly associated with the strong fundamental vibrational resonances of polarized (polyatomic) molecules that have distinctive spectral fingerprints^2,3^. Triggered by a tremendous number of applications, such as semiconductor metrology and inspection^4^, pump-probe spectroscopy^5,6^, pollution monitoring^7^, optical coherence tomography and imaging^8,9^; fiber-based laser sources capable of covering the electromagnetic spectrum from DUV (wavelengths <350 nm) up to mid-IR have attracted the scientific attention of several research groups around the globe. One of the most promising routes towards development of ultra-broad bandwidth sources is SC generation. The main nonlinear effects and physics behind SC generation have been studied extensively in the past few decades, rendering SC generation to be a well-established and matured technology^10,11^.

Most reports on SC generation in the literature are based on solid-core silica photonic crystal fibers (PCF) where the microstructured cladding of the fiber allows tailoring of the group velocity dispersion (GVD), which is an important property that greatly influences the nonlinear effects^11^. Despite silica solid-core PCF-based SC sources now being commercially available, they can only operate in a limited transmission range around 350–2300 nm due to the limited transparency window of the silica material^12^. In 2015, Jiang *et al*. reported for the first time the fabrication of a fluoride (ZBLAN) glass-based solid-core PCF with high air-filling fraction and they demonstrated a broad SC spanning more than three octaves in the spectral range 200–2500 nm^13^. However, fabrication of ZBLAN-based PCF remains very challenging, due to the very steep viscosity–temperature profile of the glass even for the fabrication of conventional step-index fibers. In order to overcome the limitations imposed both by the fiber material and fabrication difficulty, a relatively new research subfield emerged within nonlinear fiber optics, where gas-filled hollow-core PCFs are used instead of solid-core fibers^14–16^.

Nonlinear optics in hollow-core fibers and capillaries is certainly not a new field. For example, in 1970 E. Ippen demonstrated backward stimulated Raman scattering in a liquid-filled glass capillary fiber^17^. Since then, gas-filled large bore capillaries have been used as a standard approach for nonlinear applications, such as pulse compression^18^ and high-harmonic generation^19^. With the invention of hollow-core PCFs, new opportunities appeared because it became possible to have a low-loss waveguide with much smaller core diameters (on the order of few micrometers) compared to larger capillary bore sizes, this allows for the initiation of nonlinear effects through light-gas interaction at much lower pulse energies compared to capillaries^14,20^. Hollow-core PCFs and in particularly HC-ARF are divided into two main categories based on their geometrical structure: Kagome and negative-curvature^21^. The latter is a simplified form of the former which has lately received a lot of scientific attention from the fiber-optic community because it offers flat transmission spectrum, guidance in the mid-IR region, and reduced complexity from a fabrication point of view^16,21^. Nevertheless, both fiber structures have been extensively reported in the literature for gas-based experiments. The main reasons are because they offer a weak anomalous dispersion which compensates the normal dispersion of the filling gas, but more importantly they can tolerate extremely high terawatt levels of peak power due to low modal overlap with the silica cladding. Many impressive feats have been demonstrated in hollow core fibers, such as tunable and broadband DUV/Vacuum UV light generation by tuning the gas pressure inside the fiber^14,15,22–26^, four-wave mixing^27^, pulse compression in the mid-IR^28^, Raman effects^29–31^ and multistage generation of extreme UV by tapering of HC-ARF^32^. Most of the experimental papers so far report on pumping a gas-filled HC-ARF in the visible and near-infrared regime close to the zero-dispersion wavelength (ZDW) of the fiber. Recently, Cassataro *et al*. reported that pumping a Krypton-filled HC-ARF under 18 bar pressure at 1.7 μm using an optical parametric amplifier is enough to generate SC from 270 nm up to 3.1 μm with a total output energy of 4 μJ^33^. Köttig *et al*. have also demonstrated the possibility of generating mid-IR DWs (3–4 μm) by pumping in the near-IR (1030 nm) but with relatively low power spectral density (PSD)^22^. However, to the best of our knowledge there has been no experimental research carried out with pumping a single gas-filled HC-ARF with ultrashort mid-IR pulses generating a broadband SC spanning from DUV to mid-IR.

This work reports on the first experimental demonstration of multi-octave SC generation spanning from DUV to mid-IR in a gas-filled HC-ARF pumped directly in the mid-IR region at 2460 nm using a tunable optical parametric amplifier (OPA) system. By coupling 100 fs, 20 μJ pulses into a specially designed argon (Ar) filled HC-ARF under 30 bar pressure, soliton self-compression dynamics enabled broadening from 200 nm to 4000 nm. Furthermore, it was experimentally demonstrated how the pulse energy and the pressure have a crucial role in the mid-IR spectral broadening and emission of DWs in the DUV.

## Experimental and Theoretical Background

### Experimental configuration

**Figure 1:**
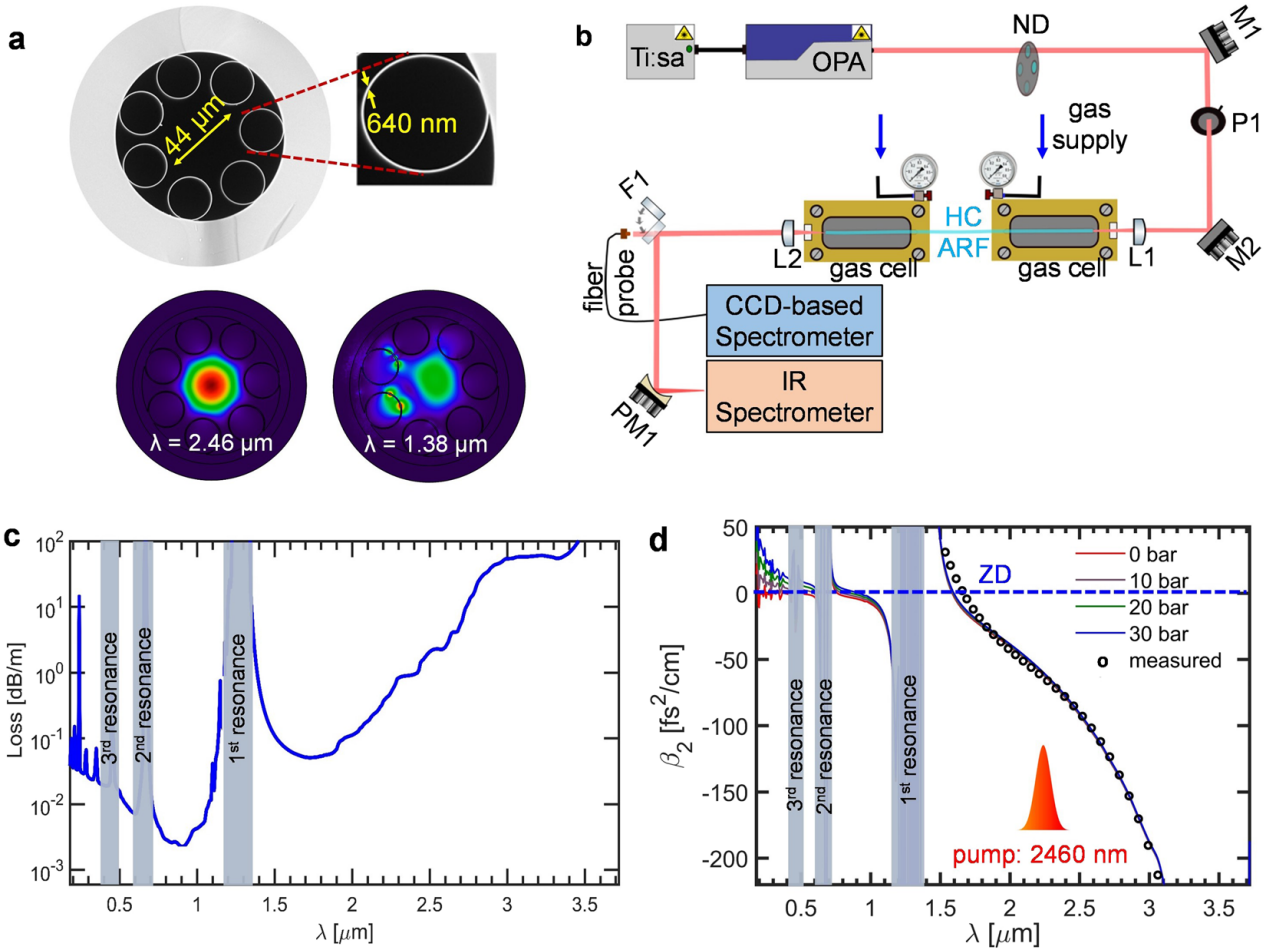
(**a**) SEM image of the HC-ARF (top panel); and mode-field profile at 2460 nm (pump wavelength) and 1380 nm (1^st^ resonance) (bottom panel). The fiber has a core diameter of ~44 μm, an average capillary diameter 25 μm, and average wall thickness of 640 ± 50 nm. (**b**) Experimental setup for SC generation in gas-filled HC-ARF. The components of the set up are: neutral density filter (ND), protected silver mirrors (M1-2), linear film polarizer (P1), CaF_2_ plano-convex lenses (L1-2), flip mirror (F1), and a gold coated parabolic mirror (PM1), 30 cm of HC- ARF. (**c**) Calculated losses (blue) for the HC-ARF including contributions from surface scattering, mode confinement, and silica material loss. The grey shaded regions show the first three resonant bands. (**d**) Calculated GVD for the HC-ARF at various levels of Ar gas pressure (solid lines). The measured dispersion of the fiber in the mid-IR is shown in circles for comparison (measured at ambient room conditions).

The fiber consists of a hollow core surrounded by seven non-touching silica capillaries with wall thickness of ~640 nm forming a core with diameter of ~44 μm, as shown in the scanning electron microscopy (SEM) image of Fig. 1(a). The calculated fundamental mode profile at 2460 nm (pump wavelength) and at the first resonance (1380 nm) is also shown in Fig. 1(a) The 30 cm long HC-ARF was suspended between the two gas cells, equipped with 5 mm CaF_2_ windows to allow coupling at the input and collimation at the output. Figure 1(b) shows the experimental setup used in this work. A Ti:sapphire amplifier was used to pump an OPA with a few milijoule pulse energy to generate the mid-IR pump. The mid-IR pump was linearly polarized, 100 fs pulses at 1 kHz repetition rate with a central wavelength of 2460 nm and its power was controlled by neutral density (ND) filters and by rotating a nanoparticle linear film polarizer (P1). The fiber output power was measured using a thermal power meter, and the beam near-field profile was imaged using a visible and near-IR CCD camera. The output spectrum was measured from 183–1100 nm using a fiber-coupled UV-VIS CCD array spectrometer, and the spectrum from 1000–5000 nm was measured using a scanning spectrometer directed by a flip mirror. Details of the experimental setup and fiber fabrication is provided in Methods section.

The insertion loss of the fiber and CaF_2_ optics was 4.2 dB (38% transmission) for a central pump wavelength of 2460 nm. This relatively high insertion loss is attributed to mode mismatch between the fiber and the pump beam. The output power was recorded for every spectral measurement, and coupling was optimized after each change in pressure and power to maintain efficient coupling to the fundamental mode and to prevent damage of the facet.

Figure 1(c) shows the calculated total propagation loss of the HC-ARF used in the experiments which is calculated based on a finite element method (FEM) software with contributions from the material (silica), mode confinement, and surface scattering loss. As it can be seen from Fig. 1(c), the HC-ARF transmits light over a broad range of wavelengths with the first anti-resonant window spanning from ~1400 nm to ~4000 nm (indicated by the 1^st^ resonance at around 1380 nm in Fig. 1(c)). Higher order anti-resonant windows allow for light to propagate even in the DUV range close to the bandgap of fused silica. The refractive index used in the calculations was based on the Sellmeier equation found in^34^, which is valid from 210 nm up to 3.7 μm. However, it is important to mention that careful consideration of the silica refractive index is required for wavelengths less than 210 nm due to strong electronic bandgap absorption^35^. To this end, the measured refractive index data of silica from^35^ was used to calculate the GVD and confinement loss for wavelengths less than 210 nm. Figure 1(d) shows the calculated GVD of the fiber from 200 nm to 3700 nm for different gas pressures from 0 to 30 bar. When the pressure of the gas in the fiber is increased it counterbalances the anomalous dispersion of the fiber, and as a result the ZDW shifts towards longer wavelengths and the nonlinear refractive index n_2_ increases, as it has been extensively described elsewhere^14–16^. In order to verify the modelling, the GVD of the HC-ARF was also measured at ambient room pressure (1 atm) and the dispersion profiles are in very good agreement, thus confirming the accuracy of the calculations.

### Modeling and theory

Single-mode and single polarization optical pulse propagation in the gas-filled HC-ARF was studied using a unidirectional field equation which also accounts for plasma effects, expressed as^36–38^:

1 $${\partial }_{z}E(z,\omega )=i(\beta (\omega )-\frac{\omega }{{v}_{p}}+i\frac{\alpha }{2})E(z,\omega )+i\frac{{\omega }^{2}}{2{c}^{2}{{\varepsilon }}_{0}\beta (\omega )}F({P}_{NL}(z,t)),$$

where *z* is the propagation distance along the fiber, *t* is the time in a reference frame moving with the pump group velocity *v*_*p*_, *E* (*z*,*ω*) is the electric field in the frequency domain, *ω* is the angular frequency, *α*(*ω*)is the linear propagation loss of the fiber, *c* is speed of light in vacuum, *β*(*ω*) is the propagation constant, and *F* represents the Fourier transform. *P*_*NL*_(*z*,*t*) is the nonlinear polarization calculated by^37,38^ ${P}_{NL}(z,t)={\varepsilon }_{0}{\chi }^{(3)}E(z,t)+{P}_{ion}(z,t),$ The first term is the Kerr effect, where *ε*_0_ is vacuum permittivity, and χ^(3)^ is the third-order nonlinear susceptibility of argon. The second term describes the nonlinear polarization due to molecular ionization calculated as^22,37–39^:

2 $${P}_{ion}(z,t)={\int }_{-\infty }^{t}\frac{\partial \rho }{\partial {t}^{\text{'}}}\frac{{I}_{p}}{E(z,{t}^{\text{'}})}d{t}^{\text{'}}+\frac{{e}^{2}}{{m}_{e}}{\int }_{-\infty }^{t}{\int }_{-\infty }^{{t}^{\text{'}}}\rho (z,{t}^{\text{'}\text{'}})E(z,{t}^{\text{'}\text{'}})d{t}^{\text{'}\text{'}}d{t}^{\text{'}},$$

where *ρ* is density of free electrons, *m*_*e*_ and *e* are the mass and charge of an electron, and *I*_*p*_ is gas ionization energy. In our calculation, the nonlinear refractive index (*n*_2_) of Ar was assumed to be wavelength independent^36,40^ and the Raman contribution from silica was neglected due to the very low light-glass overlap (≪1%) in HC-ARF^36,41,42^. For gas-filled fibers, an optical pulse intensity in the range of 100 TW/cm2 corresponds to a Keldysh parameter, *p*_k_ ≤ 1^43,44^. In our gas-filled system, the peak intensity reaches an estimated 190 TW/cm2 confirming *p*_k_ ≪ 1, in which case tunneling ionization dominates over multiphoton ionization^36^. Experimental results also suggest that the tunneling model is in good agreement with the experiments, even with *p*_k_ ≃ 1^45,46^. Therefore, to calculate the free electron density, quasi-static tunneling ionization was chosen based on the Ammosov, Delone, and Krainov (ADK) model which is described in^43^.

**Figure 2:**
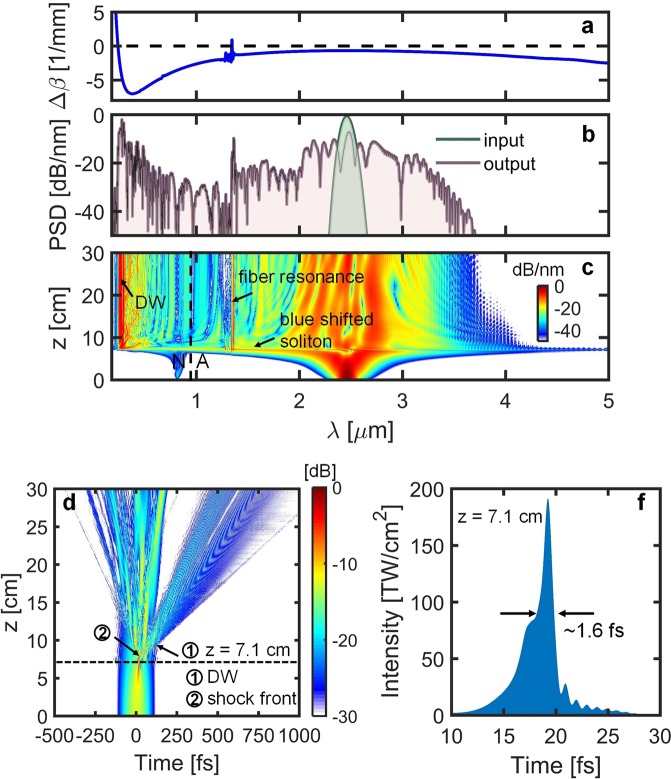
Numerically simulated (**a**) propagation constant mismatch (Δβ) between the soliton and dispersive waves, (**b**) normalized PSD at z = 0 and z = 30 cm, (**c**) spectral evolution (normalized to the peak intensity), (**d**) temporal evolution in dB scale, and (**e**) intensity at the maximum compression point at z = 7.1 cm for a ~44 μm core HC-AR fiber under 30 bar of Ar gas, pulse energy 8 μJ, pulse duration 100 fs, and pumping in the anomalous dispersion regime at 2460 nm. N: normal dispersion regime, and A: anomalous dispersion regime. The fiber dispersion was calculated using finite-element modeling.

The evolution of the spectral and temporal profile in a 44 μm core HC-ARF filled with 30 bar Ar and pumped in the anomalous dispersion regime at 2460 nm with 100 fs Gaussian pulses having 8 μJ energy are shown in Fig. 2(c,d), respectively. After propagating 7.1 cm, the pulse experiences strong soliton self-compression down to ~1.6 fs with peak intensity ~190 TW/cm^2^ due to the combined action of self-phase modulation (SPM), anomalous dispersion, and optical shock effects^36,38^ (see Fig. 2(d)). At the maximum temporal compression point, an efficient dispersive wave (DW) is emitted at 278 nm. To confirm that this is in fact a DW, the propagation constant mismatch Δβ with respect to the pump was calculated as a function of wavelength, as shown in Fig. 2(a), using the following expression^22,38^

3 $${\rm{\Delta }}\beta (\omega )\approx \beta (\omega )-({\beta }_{0}+(\omega -{\omega }_{0}){\beta }_{1}+\gamma N{P}_{0}\omega /{\omega }_{0}-\frac{{\omega }_{0}\rho {\omega }_{0}}{2{n}_{0}c{\rho }_{cr}\omega }),$$

where, *β*_0_ is the propagation constant and *β*_1_ = 1/*v*_*g*_ is the inverse group velocity at *ω*_0_, *P*_0_ is the pump peak power, *N* is the soliton order, *γ* = *n*_2_/c*A*_eff_ is the fiber nonlinear coefficient, *A*_eff_ is the effective mode area of the fiber, *ρ* is the plasma density and *ρ*_cr_ is the critical plasma density at which plasma becomes opaque^22,38^. In the spectral profile, a narrow band emission peak was found at the resonance wavelength of ~1360 nm despite the high loss at this wavelength. This feature at the vicinity of the anti-crossing is a consequence of the phase-matching curve crossing *Δβ* = 0 resulting in four-wave mixing and the DW emission, as recently reported in ref.^47,48^. It can be seen from Fig. 2(c) that a blue shifted soliton is found at the maximum temporal compression point because the high peak intensity is sufficient to ionize the gas and form a plasma (see Fig. 2(c)). Due to the plasma formation, the spectrum broadens mainly to the blue side and a 4.3-octave-wide SC is generated covering 200 nm to above 4000 nm. At the soliton self-compression stage, the average nonlinear refractive index change becomes negative which in turn provides a positive phase shift and a shift towards the blue side of the spectrum, whereas the index change remains positive before and after the self-compression^49^. It is important to emphasize that these simulations assume a spatially invariant fundamental mode. It is known that ultra-high peak power can initiate beam self-focusing above the critical peak power ${P}_{cr}={\lambda }_{0}/(2\pi {n}_{0}{n}_{2})$, which in turn may lead to beam filamentation depending on the balance between Kerr self-focusing and plasma defocusing^50^. These dynamics are however very sensitive to the corresponding temporal dynamics, and since both n_2_ and the GVD scale with the gas pressure, the self-focusing dynamics will depend critically on the gas pressure^50^. As a result the true spatio-temporal dynamics present a highly complex problem that requires more elaborate models to investigate, as detailed in^50,51^. The implementation of the numerical model presented here is described in ref. ^36^.

## Results and Discussion

**Figure 3:**
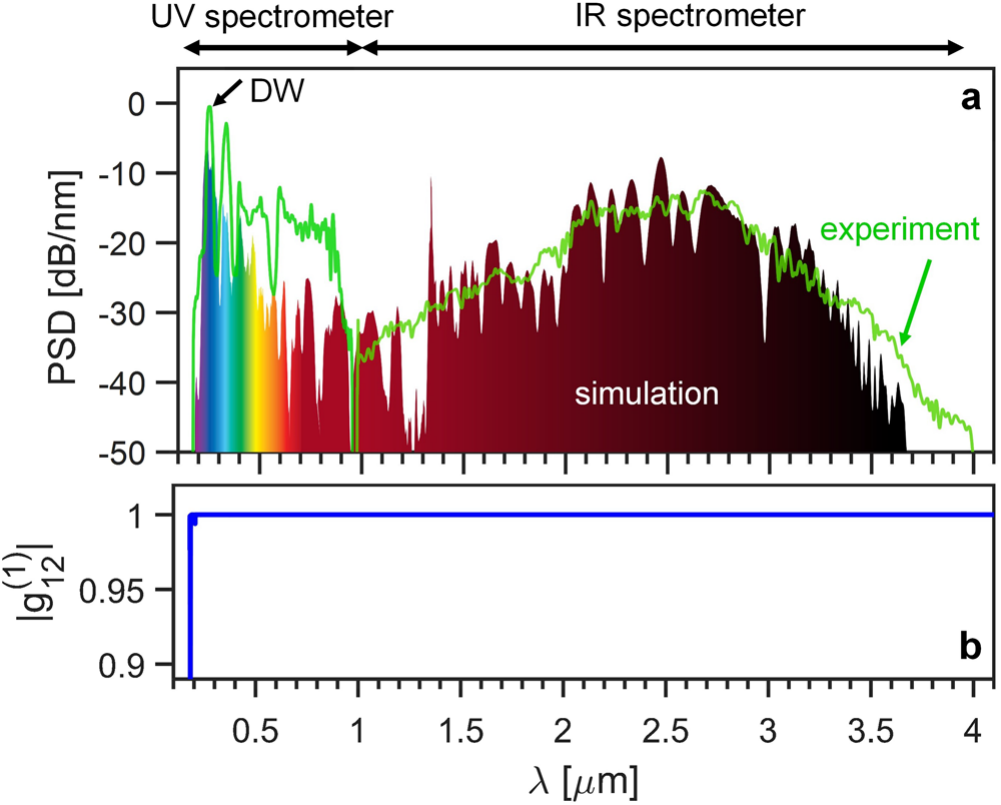
(**a**) Simulation and measurement of SC spectra obtained by pumping the HC-ARF at 2460 nm (anomalous dispersion region) with 100 fs pulses (**b**) Calculated complex degree of coherence of the generated SC.

Figure 3(a) shows the comparison of the measured and calculated SC spectra. The measured spectrum spans from 200 nm up to 4000 nm, while the simulated spectrum spans from 200 nm to 3700 nm. Before the start of the experiments, the fiber was purged several times with high purity Ar (AGA A/S) to remove any remaining atmospheric air and other impurities. The fiber length was 30 cm due to allow for practical handling of the fiber while assembling the gas-cells. At the maximum pump power of 20 mW, corresponding to a pulse energy of 20 μJ, a total of 5 mW average power was measured out of the fiber after the collimation lens. By taking into account the loss of the CaF_2_ optics, which has 94% transmission at 2460 nm and ~92% transmission on average from 200–4000 nm (source: Thorlabs) and including around 2 dB/m propagation loss at the pump wavelength, the 5 μJ output energy correspond to an estimated injected pulse energy of around 8 μJ. This estimate is corroborated by the qualitative agreement between experiments and simulations for an injected pulse energy of 8 μJ.

**Figure 4:**
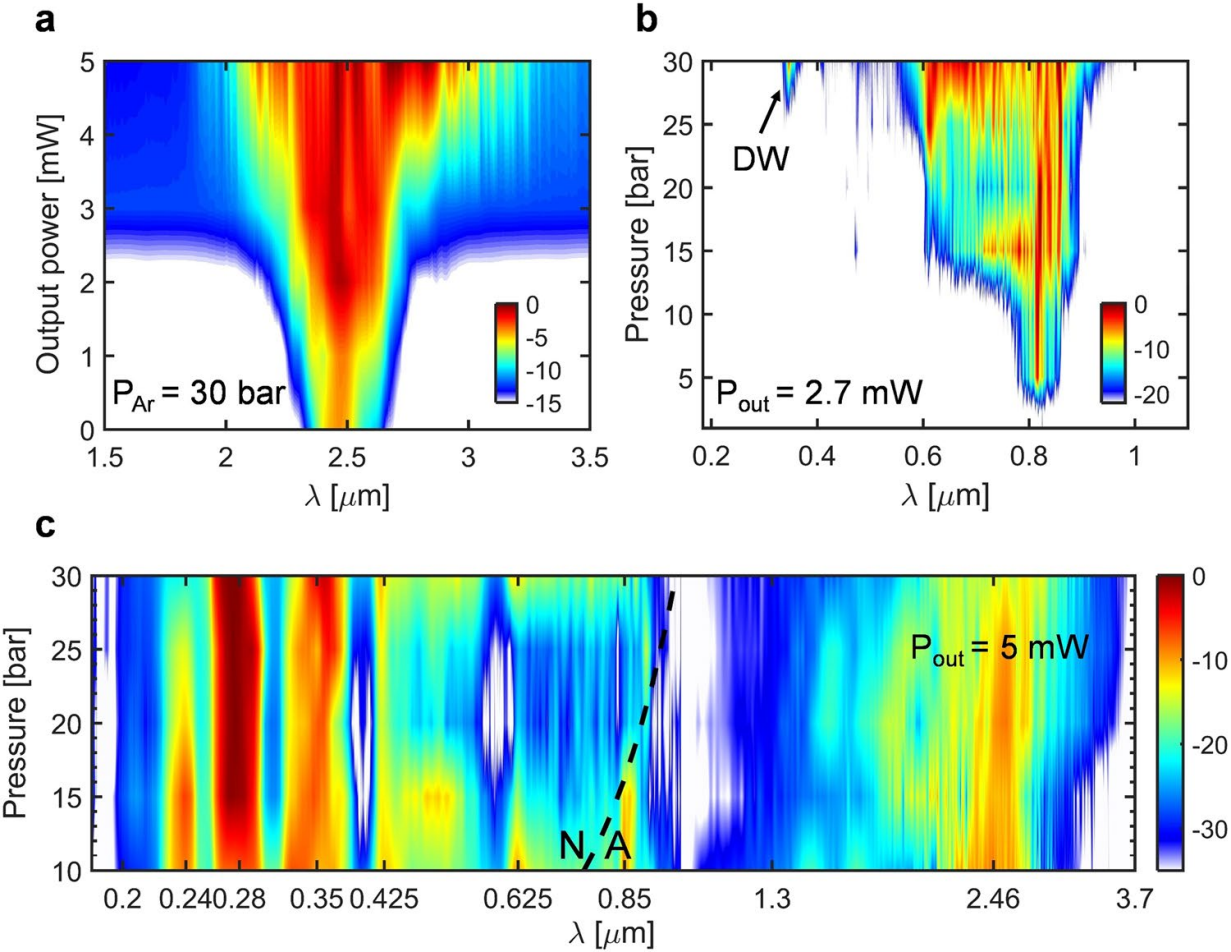
(**a**) Measured spectral evolution and DW formation in the near/mid-IR range indicating the spectrum broadening as a function of measured output power for a fixed pressure of 30 bar. (**b**) Spectral broadening and DW emission as a function of pressure in the DUV/visible. (**c**) Pressure dependent evolution of the spectrum at a fixed low power of 5 mW over the full spectrum from 10 up to 30 bar with a step of 5 bar.

Furthermore, simulations predict a very bright and spectrally coherent continuum. The coherence of light sources is very crucial for many applications such as spectroscopy and other quantitative techniques where fluctuations in intensity will result in increased noise, thereby reducing the signal to noise ratio. Raman scattering and fiber propagation losses result in additional noise terms that adds to the quantum noise, but since the HC-ARF used was designed for high transmission from UV to mid-IR and Ar is a non-polar Raman-free gas, the contributions from these effects are negligible. The calculated first-order complex degree of coherence of the SC is shown in Fig. 3(b), using the expression^23,52^:

4 $$|{g}_{12}^{(1)}|=|\frac{\langle {A}_{1}^{\ast }(\omega ){A}_{2}(\omega )\rangle }{\langle {|{A}_{1}(\omega )|}^{2}\rangle }|,$$

where the angle brackets represent an ensemble average over independent simulations while taking into account random quantum noise fluctuations based on one photon per mode model. The value of |g^(1)^_12_| indicates quality of the coherence of the SC and the coherence is perfect if |g^(1)^_12_| = 1 meaning that the electric fields have perfectly equal phase between different laser shots whereas |g^(1)^_12_| = 0 indicates random phase fluctuation from shot-to-shot. Figure 4(b) depicts that the first-order coherence of the generated spectrum is fully coherent |g^(1)^_12_| ≈ 1 under the assumption of a single spatial and polarization mode.

The main limitation of the spectral extension towards the mid-IR is the increasing propagation losses, reaching 100 dB/m at 3500 nm from which silica multiphonon absorption is the dominant loss mechanism. This was also the conclusion from the numerical simulations, as seen in Fig. 2(b). To improve the mid-IR transmission it is therefore paramount to reduce the mode overlap with the silica structure to a minimum, which can be achieved through fiber geometric modification. Figure 4(a) shows the mid-IR spectral broadening with increasing pump power at maximum constant pressure of 30 bar where the nonlinearity is high. At lower pump power (1–3 mW output power) the limited broadening observed can be attributed to mainly the effect of SPM. In this low-power regime the mid-IR broadening is found to be only weakly dependent on pressure. However, as seen in Fig. 4(b) the visible part of the spectrum was highly influenced by changes in the pressure. This is to be expected from the significant change in the GVD in the visible compared to the mid-IR. At higher power, the pressure begins to have a more pronounced effect on the mid-IR and UV-visible regime. These dynamics are shown for 5 mW output power in Fig. 4(c) using a base 2 logarithmic wavelength axis for better visibility of the UV region. Above 15 bar the long-wavelength edge starts to increase together with the energy of the DWs around 275 nm, while simultaneously diminishing the energy of the DWs around 240 nm. The increasing mid-IR broadening and 275 nm DW generation is attributed to the increasing nonlinearity, while the diminishing 240 nm DW generation is believed to be caused by the red-shifting of the ZDW. Based on the experiments and simulations, it is evident that pumping directly in the mid-IR allows for a higher fraction of energy in the near- and mid-IR compared to pumping in the near-IR^22^, while retaining efficient DW generation down in the DUV region.

In conclusion, multi-octave SC generation was demonstrated in a gas-filled HC-ARF pumped in the mid-IR region. The SC embedded in a 4.3-octave-wide spectrum spans from 200 nm up to 4000 nm when 8 μJ, 100 fs pulses are injected into the fiber filled with Ar at a pressure of 30 bar. A total measured average output power of 5 mW (at 1 kHz repetition rate) was obtained with a strong resonant DW emission at 275 nm. Finally, it was experimentally demonstrated how the pump energy and pressure increases the nonlinearity resulting in increased mid-IR spectral broadening and efficient DW emission in the DUV range. The current work constitutes an efficient route towards ultrafast source for spectroscopy both in the mid-IR molecular fingerprinting and in the DUV spectral region.

## Methods

### HC-ARF fabrication and characterization

The HC-ARF used in this experiment was specially designed for high transmission in the mid-IR region. It is fabricated through the well-known stack and draw technique, where seven capillaries of silica are stacked inside larger silica tube to form the preform. The preform is then drawn into canes which is later drawn again to attain fibers of few hundred microns in diameter. The detailed fabrication process is presented elsewhere^53^. With recent fiber design showing the possibility of achieving single mode propagation and ultralow loss in AR fibers^54^. The final stage of the drawn fiber consists of a hollow core surrounded by seven non-touching silica capillaries with wall thickness of ~640 nm forming a core with diameter of ~44 μm, as shown in the SEM image in Fig. 1(a). For fiber characterization; SEM image of cross-section of the fiber was taken and dimensions of its inner capillaries were used to numerically compute the fiber resonances. The fiber dispersion was then measured using a commercial SC source (mid-IR superK compact, NKT Photonics A/S) with spectral range from 1500 nm to 4000 nm and average power of around 100 mW.

### Gas-filling setup

The gas cells were connected with plastic tubes for gas entry and a valve for purging and gas evacuation. The fiber was mounted and fixed to a v-groove inside the gas-cell to ensure stability at high pressure and the two gas-cells were mounted on micro-translation stages and moved in parallel to avoid fiber bending while allowing for flexible input coupling. The light is free-space-focused into the fiber with a plano-convex lens of 50 mm focal length and a similar lens collimates the light at the output of the fiber. Both windows and lenses were uncoated to obtain >90% transmission from 200–5000 nm. The beam was directed to either a CCD-based detector (Oceanoptics HR2000+) or to an IR Spectrometer by using a flip-mirror as shown in Fig. 1(b), The IR spectrometer is equipped with a mercury-cadmium-telluride (MCT) detector and box-car integrator. The scanning spectrometer included an automatic long-pass filter-wheel to eliminate higher-order diffraction and the spectral response of the entire system was calibrated using a 1273 K blackbody source.

The gas used for the experiments is compressed Ar (99.998% purity), and a pressure of up to 30 bar was applied into the fiber from both gas-cells to maintain uniform pressure in the system. The pressure was varied using a manual high-pressure regulator. The measurements were stopped a few seconds after every change of pressure to ensure uniform distribution of pressure in the tubes, gas-cells and the fiber. The recorded spectrum was found to be stable over several hours of operation and no sign of solarization or silica degradation observed during the experiments.

### 4.3 Spectral data calibration, stitching, and power normalization

To estimate the energy fraction in the 275 nm DW, the UV-Vis part of the spectrum measured using the CCD spectrometer (183 nm to 1100 nm) was stitched to the mid-IR part of the spectrum measured with the MTC detector (1000 nm to 5000 nm). Since both spectrometers were intensity calibrated, the total spectrum was numerically resampled and normalized to the total measured output power to obtain a µW/nm scale. At 30 bar pressure and 5 mW output power, the integrated energy of the highest peak at 275 nm was estimated to be 1.42 µJ (28.4%). This value is higher than the 15% predicted by the simulations, and what has previously been reported in literature. Without a bandpass filter centered around 275 nm this could not be directly verified. However, to demonstrate the veracity of the spectrum in the DUV regime, bandpass filters centered at 360 nm and 380 nm were used to attest that the signal and the intensity were accurate.
